# 

**Published:** 1872-07

**Authors:** 


**PARTICULAR NOTICE.**

☞ ***THE JOURNAL will not be sent, after the present number, to any who have not renewed their subscriptions.***

☞ **Remit $3 for the current volume, commencing January, 1872.**

